# Maternal Attachment Representations during Pregnancy, Perinatal Maternal Depression, and Parenting Stress: Relations to Child’s Attachment

**DOI:** 10.3390/ijerph19010069

**Published:** 2021-12-22

**Authors:** Cristina Sechi, Laura Elvira Prino, Luca Rollé, Loredana Lucarelli, Laura Vismara

**Affiliations:** 1 Department of Education, Psychology, Philosophy, University of Cagliari, 09123 Cagliari, Italy; cristina.sechi@unica.it (C.S.); llucarelli@unica.it (L.L.); 2 Department of Philosophy and Education Sciences, University of Torino, 10124 Torino, Italy; lauraelvira.prino@unito.it; 3 Department of Psychology, University of Torino, 10124 Torino, Italy; l.rolle@unito.it

**Keywords:** maternal attachment, perinatal depression, parenting stress, child attachment

## Abstract

Background: This paper aimed to explore the associations between maternal representations of attachment evaluated during pregnancy, pre and postnatal maternal depression, parenting stress and child’s attachment at 15 months after childbirth. Methods: Mothers (*n* = 71), and their infants participated in a longitudinal study of maternal attachment, pre and postnatal depression, parenting stress and child attachment. Adult Attachment Interview (AAI) was conducted between 24 and 26 weeks of pregnancy (Time 1), depression was assessed using the Edinburgh Perinatal Depression Scale (EPDS) (at Time 1 and 6 months after childbirth, i.e., Time 2), parenting stress was assessed using the Parenting Stress Index—Short Form (PS-SF) (at Time 2) and the Strange Situation Procedure (SSP) at child’s 15 months of age (Time 3). Results: Free-autonomous maternal classification of attachment increases the likelihood of secure child classification in her offspring, while decreases that of avoidance and ambivalence. Insecure maternal representation of attachment evaluated during pregnancy and higher levels of parenting stress at six months after childbirth was associated with higher rates of infant insecure attachment at 15 months. Conclusions: Our study validates the importance of considering maternal representations of attachment crucial in determining the quality of the caregiving environment, thereby the healthy development of children, despite the presence of other contextual risk.

## 1. Introduction

Maternal depression is a common disorder among women [1,2]. It has been established that about one in nine women suffer from postpartum depression; consequently, many children will experience maternal depression in their first year of life [3].

Maternal depression is associated with sadness, lethargy, helplessness, irritability that leads to either a withdrawn, unresponsive or hostile, intrusive interactive style with their infant [4,5,6,7]. These manifestations have proven to compromise the development of a secure attachment relationship between mother and infant [8,9,10,11]. Secure attachment is fostered by a caregiver who is sensitive and responsive to their infant’s signals of distress. Eventually, this relationship favors the child’s behavioral and emotional functioning [12,13]. For such reason, screening for depression in postpartum women through a self-reported questionnaire should be a major goal to support perinatal mental health [14].

Maternal depression is also associated with parenting stress; indeed, postpartum depression has been considered among the strongest predictors of parental stress both in terms of biological outcomes and caregiving behaviors [15,16,17]. In the current study, we focus on parenting stress that may be defined as the perception of incongruence between the demands of parenting and parental accessible resources [18,19]. Parenting stress correlates with lower maternal warmth and sensitivity [20,21]; consequently, it is also considered connected to the quality of attachment both in the infants and in their parents [22,23,24,25].

Maternal depression and parenting stress can therefore impair the child’s development since the mothers who are experiencing these conditions appear to have problems in interacting and being emotionally available to their child [26].

Overall, several scholars indicated the close and complex links among maternal depression, parenting stress, and mother-child attachment [17,27,28,29,30,31], yet, findings are inconsistent [32]. Hence, our purpose is to elucidate the relation among maternal self-reported depressive symptoms, parenting stress and insecure mother-child attachment. We suggest that the quality of maternal attachment representations may influence the power of such associations.

In particular, this paper explored the following aims in a group of first-time Italian mothers:

1. The associations between maternal representations of attachment evaluated during pregnancy, pre and postnatal self-reported depressive symptoms, parenting stress and child’s attachment at 15 months after childbirth;
2. How child attachment, assessed at 15 months of the child, can be predicted by each of the pre and postnatal variables investigated.

Based on the reported research, a relationship among mother’s attachment representations, self-reported depressive symptoms and parenting stress and her child’s attachment was expected. It was hypothesized that perinatal depression and parenting stress would be associated with both an insecure maternal representation of attachment during pregnancy and an insecurely attached child. Besides, we hypothesized that the maternal representation of attachment during pregnancy, maternal self-reported depressive symptoms, and parenting stress would impact on child–caregiver attachment at 15 months.

## 2. Materials and Methods

### 2.1. Participants

The participants were part of a longitudinal study on maternal depression in first-time mothers and the development of their children’s affective regulation systems. These women were recruited from neonatology units and family healthcare services in Cagliari and Turin (Italy). Participation was voluntary, and no incentives were offered for participation. Data were collected at 7 months of pregnancy (Time 1) and when the children were 6 and 15 months of age (Time 2 and Time 3 respectively). All mothers were European Caucasian women with no previous history of pregnancy, stillbirth, miscarriage, or neonatal death. Out of 89 mothers who participated in this study, twenty-five were lost to follow-up (18 mothers had dropped out at Time 2, and 7 mothers had dropped out at Time 3). Therefore, a total of 71 mothers and their children (37 males, 34 females) completed all three assessments. Mothers not retained in the study did not differ from the remaining sample on maternal age, education, and child gender *p* > 0.05. The average age of the mothers was 34.3 (SD = 4.75), with a range of 20–45 years. Of these mothers, 17% had elementary-school degree, 28% had high-school degree, 48% had college degrees, and 7% had PhDs. All the mothers were married or lived with their partners. The median income of these mothers fit the national middle working-class socio-economic status, according to the classification reported by the Italian National Statistics Institution (2016).

### 2.2. Measures

Adult Attachment Interview (AAI; [33]): The AAI is a 20-questions semi-structured interview that evaluates the coherence of the narration of an individual about their childhood experiences with their caregivers. The interviews were audio-taped, transcribed verbatim and then coded by two certified Italian AAI coders using the system developed by Main and Goldwyn [34]. Specifically, AAI transcripts were first rated on a series of 9-point scales capturing the probable experience of the interviewee with her parents during childhood (e.g., loving, rejection, neglecting), as well as the state of mind concerning her attachment experiences (e.g., idealization, lack of memory, anger). Finally, the transcripts are assigned to one of the four main categories: free-autonomous (F) if mothers valued attachment experiences and were coherent, dismissing (Ds) if mothers were actively derogating or dismissive about their attachment experiences, enmeshed/preoccupied (E) if they appeared currently overinvolved about their past attachment experiences, and unresolved/disoriented (U/d) in the presence of lapses of reasoning and discourse concerning experiences of abuse or loss of attachment figures. In the presence of a global lack of organization throughout the whole interview, mothers were assigned to the ‘cannot classify’ (CC) category [35]. These transcripts were assigned to the best fitting ‘forced’ category. Coders were blind to other maternal and child evaluations. Inter-rater reliability was assessed on 28% of the transcripts, yielding an 83% agreement with a Cohen’s kappa = 0.88 (*p* < 0.001). 

Edinburgh Postnatal Depression Scale (EPDS; [36]): The EPDS is a self-report questionnaire including ten items addressing depression symptoms occurring within the earlier 7 days. The overall score is computed by adding items on a 4-point Likert scale. Higher scores reflect more depression symptoms. In the current study, the internal consistency coefficient was α = 0.90 at Time 1 and α = 0.85 at Time 2.

Parenting Stress Index—Short Form (PSI-SF; [37]): The PSI-SF is a self-report instrument that measures stress specifically associated with parenting. It consists of 36 statements within the past week. The mother rates each item on a 5-point Likert scale ranging from “strongly agree” to “strongly disagree.” Mothers who obtain a Total Stress score above a raw score of 90 are considered as experiencing clinically significant parenting stress. The PSI-SF yields three subscales (parental distress, parent-child dysfunctional interaction, and difficult child) and a summary index of total stress. This was the only summary index applied in this study. Higher scores reflect more parenting stress. In the current study, the internal consistency coefficient was α = 0.89 at Time 1 and α = 0.87 at Time 2.

Strange Situation Procedure (SSP; [38]): It consists of eight three-minute episodes, including two separations from the mother, each followed by a reunion. The SSP was conducted in a one-way mirror laboratory equipped with cameras. The two reunions (episodes 5 and 8) are the focus of experts’ rating of attachment behaviors. Two certified Italian SSP raters reviewed the video and rated four infant attachment behaviors in the two reunions: proximity-seeking (approaching mother), contact-maintenance (staying close to mother), resistance (to contact with mother), and avoidance (ignoring or moving away from mother). Based on the Ainsworth et al. [38] coding system, children were classified as secure (B) if they actively sought contact with their mother and were readily reassured by her, insecure-avoidant (A) if they avoided their mother on reunion, and insecure-ambivalent (C) if they showed angry or passive behavior toward their mother on reunion and were not comforted by her presence. 

Coders were blind to other maternal evaluations. Inter-rater reliability was assessed on 30% of the infant–mother videotapes, yielding an 84% agreement with a Cohen’s kappa = 0.74 (*p* < 0.01). Disagreements were resolved through exhaustive consultations leading to consensus.

### 2.3. Procedure

The present research project obtained approval from the University Ethics Committee of the Department of Pedagogy, Psychology and Philosophy, of the University of Cagliari (Protocol n°2/2013; 5 February 2013). Prior to data collection, the mothers received complete information concerning the research procedures and provided their written informed consent to participate in the study.

The longitudinal study included three assessments, in which different types of instruments were administrated at the Department of Pedagogy, Psychology and Philosophy, University of Cagliari and at the Department of Psychology, University of Turin (Italy). Specifically, between 24 and 26 weeks of pregnancy (Time 1), mothers were administered the Adult Attachment Interview. All mothers independently filled out the EPDS. Six months after childbirth (Time 2), mothers were asked to fill out the EPDS and the PSI-SF. The 15-month follow-up (Time 3) involved a videotaped assessment of the child’s attachment using the SSP. All attachment transcripts and videotapes were classified by trained research assistants.

### 2.4. Data Analysis

SPSS 21.0 was used for statistical analyses. Preliminary analyses explored distributions of maternal and child attachment classifications. Correlation’s analysis was used to assess the association between demographic variables, mother and child attachment classifications, depression symptoms and parenting stress.

Independent samples *t*-test was conducted to examine whether maternal perinatal depressive symptoms and parenting stress differed for mothers classified as secure and those classified as insecure as well as for children classified as secure and those classified as insecure.

Finally, to determine whether child’s attachment classifications mainly reflected the mother’s attachment and/or was influenced by perinatal depressive symptoms and parenting stress, a logistic regression equation was tested.

Research manuscripts reporting large datasets that are deposited in a publicly available database should specify where the data were deposited and provide the relevant accession numbers. 

## 3. Results

### 3.1. Adult Attachment Interview Classifications

The distributions were as follows: 49 mothers (69%) were given a primary classification of secure (F). The insecure mothers group comprised: 10 mothers (14%) dismissing (Ds); 6 mothers (8.5%) enmeshed-preoccupied (E); 4 mothers (5.6%) unresolved/disoriented (U/d) and 2 mothers (2.8%) ‘cannot classify’ (CC). Within the unresolved/disoriented and ‘cannot classify’ groups, 3 mothers had a forced classification as dismissing and 3 mothers had a forced classification as preoccupied. All two ‘cannot classify’ cases had a forced classification as enmeshed-preoccupied.

### 3.2. Strange Situation Classifications

The distributions were as follows: 52 children (73.2%) were given a primary classification of secure (B). The insecure children group was constituted as follows: 7 children (9.9%) avoidant (A) and 12 children (16.9%) ambivalent (C).

### 3.3. Relations between AAI Classifications and Strange Situation Classifications

Table 1 shows the frequencies for the three-way comparisons between mothers’ classification on the AAI and the child’s SSP classification. Autonomous adult classification increases the likelihood of secure child classification while reducing that of avoidant and ambivalent. Specifically, 58% of mothers classified as autonomous (F) had children classified as secure (B), 4.2% of mothers classified as dismissing (Ds) had children classified as avoidant (A), and 2.8% of mothers classified as enmeshed-preoccupied (E) had children classified as ambivalent (C), χ^2^ = (3, N = 71), 12.548, *p* < 0.05.

### 3.4. Relationships among Pre and Postnatal Depressive Symptoms, Parenting Stress, Maternal and Child Attachment

For the purpose of this analysis, dismissing, preoccupied, unresolved/disoriented, and cannot classify mothers’ groups were combined into a single, insecure group. Similarly, the avoidant and ambivalent child groups were combined into a single insecure group. Analyses were first conducted to determine if any demographic variables were associated with mothers’ prenatal attachment, pre and postnatal depressive symptoms, parenting stress and children’s attachment at 15 months. The following demographic variables were tested: maternal age, maternal education, and child gender. No demographic variable was significantly related to mothers’ prenatal attachment, pre and postnatal depressive symptoms, parenting stress, and children’s attachment.

The intercorrelations among the study variables are shown in Table 2. As predicted, depressive symptoms at Time 1 and Time 2 and parenting stress at Time 2 were positively related to maternal insecure classification during pregnancy. Depressive symptoms and parenting stress at Time 2 were positively related to child insecure classification.

To examine whether mothers classified as insecure had higher levels of pre and postnatal depressive symptoms and postnatal parenting stress compared with mothers classified as secure, independent samples *t*-test were conducted. The mean scores of depressive symptoms measured by the EPDS and the parental stress measured by PSI-SF, for the secure and insecure mother groups, are shown in Table 3. Insecure mothers reported significantly higher scores on the EPDS scores both Time 1 and Time 2 than secure mothers; *t* = −2.71(69) *p* < 0.01, *d* = 0.68; *t* = −3.57 (69) *p* < 0.01, *d* = 0.91 respectively. Insecure mothers reported significantly higher levels of parenting stress at Time 2 than secure mothers; *t* = −2.71(69) *p* < 0.01, *d* = 0.58.

Finally, independent samples t-tests were conducted to examine whether depressive symptoms at Time 1 and Time 2 and parenting stress at Time 2 differed for infants classified as secure and those classified as insecure. The mean scores of depressive symptoms measured by the EPDS and the parental stress measured by PSI-SF, for the secure and insecure child groups, are shown in Table 3. There was no significant difference in maternal depressive symptoms at Time 1 for children classified as secure and those classified as insecure, *t* = −1.95(69) *p* = 0.06, *d* = 0.49. However, children classified as insecure had mothers with higher levels of depressive symptoms at Time 2 compared with infants classified as secure, *t* = −2.33(69) *p* < 0.05, *d* = 0.62 as well as children classified as insecure had mothers with higher levels of parenting stress at Time 2 compared with infants classified as secure *t* = −3.26 (69) *p* < 0.01, *d* = 0.78.

In order to test the impact of maternal representations of attachment during pregnancy on child attachment, a logistic regression analysis was conducted including maternal attachment classification (secure vs. insecure), EPDS scores at Time 1 and Time 2 and PSI-SF scores at Time 2 as predictors, child’s attachment (secure vs. insecure) as outcome.

The model as a whole explained 27.6% (Nagelkerke R squared) of the variance in child attachment status. Moreover, the Hosmer and Lemeshow test indicated a satisfactory fit for the model (χ^2^ = 5.54, df = 8; *p* = 0.69). As shown in Table 4, only maternal representations of attachment during pregnancy and parenting stress at Time 2 made a statistically significant contribution to the model. No significant contributions were found for depressive symptoms at Time 1 and Time 2.

## 4. Discussion

The present study shows that maternal self-reported depressive symptoms, parenting stress, and attachment security affect the child’s attachment. In particular, the free-autonomous maternal classification of attachment increases the likelihood of secure child classification in her offspring, while it decreases that of avoidance and ambivalence, in line with previous findings [39,40]. Certainly, these mothers are capable of acknowledging the relevance of their child’s attachment needs, thereby fostering the creation of a secure base. However, albeit the proved association between mother and child attachment security, several scholars observe inconsistency in the statistical power of such association, that may be explained by the contribution of other variables [41,42]. For such reason, the current study included both maternal self-reported depressive symptoms and parenting stress as potential, relevant involved dimensions in the explanation of the relation between maternal and child’s attachment. In fact, both postnatal self-reported depressive symptoms and parenting stress were positively related to maternal insecure classification during pregnancy and to their child’s insecurity at 15 months of age. Certainly, mothers who self-report depressive symptoms are less responsive, loving and sensitive to their children [43,44], even in the presence of mild depressive symptoms [45]. However, we found a positive association between prenatal depressive symptoms and maternal insecurity, while no association with child’s insecurity was detected. We may suppose that prenatal depressive symptoms may reflect the mother’s uncertainty and low trust on her caregiving abilities during the transition to parenthood that may characterize insecurely attached mothers, whereas transient, prenatal depressive symptoms are not per se sufficient to impact the children’s attachment [10].

Furthermore, the ability to adjust to the parenting role seems to be associated with maternal secure attachment [46,47]. Undeniably, the representation of the self and others as assessed by the Adult Attachment Interview are linked to parental mental states and caregiving behaviors towards the child [48,49]. So, in the presence of maternal attachment insecurity, it is likely that mothers experience higher levels of parenting stress facing their child’s demands and the challenges of caregiving [24].

In order to accurately measure the impact of maternal representations of attachment during pregnancy on child attachment, we conducted a logistic regression analysis, including maternal attachment classification (secure vs. insecure), prenatal and postnatal self-reported depressive symptoms and postnatal parenting as predictors, and child’s attachment (secure vs. insecure) as the outcome. 

The model shows that only maternal representations of attachment during pregnancy and postnatal parenting stress made a statistically significant contribution to the model. No significant contributions were found for pre nor postnatal depressive symptoms.

These results highlight the power of mothers’ attachment representations in predicting their child’s attachment. Definitely, an insecure internalized model of one’s own past attachment experiences may stably undermine the mothers’ capacity of caregiving, who distrust in others’ availability and in their own abilities; this is especially true if we consider the delicate, stressful changes that accompany the transition to parenthood [50].

Also, parenting stress seems to play a crucial role. Several studies have shown that maternal and family stress is related to child’s attachment insecurity [51,52,53]; however, our study focuses specifically on parenting stress, which is characterized by feelings that parenting is onerous and difficult [54,55]. We may assume that parenting stress may be associated with more negative, inconsistent caregiving behaviors [19,24,56], that in turn contribute to poorer attachment relations with the mother. Future studies are needed to verify this outcome, which has been too little investigated, despite its relevance in mothers’ lives.

Findings with respect to self-reported depressive symptoms, instead, are in line with other studies; even if children of depressed mothers are at a higher risk to develop an insecure attachment, not all children will [11,41,57,58]. Stronger associations are present when maternal depression is severe, chronic, and diagnosed by expert clinicians [44,59,60,61,62], whereas, in our sample, mothers reported a low level of depressive symptoms, on average, during the postpartum period. Thus, our findings support the need to consider both specific and cumulative risk factors in the understanding of the quality of the mother–child relationship and of the child’s functioning.

### Limitations

Some limitations need to be considered. First, the participants are all highly educated white European women; therefore, our results cannot be generalized to other SES groups or other cultures. On the other hand, this characteristic allows to reduce the influence of confounding variables typical of high-risk sample.

The second limitation regards the statistical power of the study. Surely, the number of participants did not allow to differentiate among the different attachment classifications, which can provide important additional information on the different developmental trajectories.

Further, the study administered self-reports concerning depression and parenting stress; hence, the mothers may have answered in a socially desirable way or may have been low on their capacity to acknowledge their own psychological experience. In addition, methodological issues may be raised by the application of cutoff scores to detect self-reported depressive symptoms; specifically, lower sensitivity (i.e., the number of women who are correctly diagnosed as depressed) and specificity (i.e., the number of women who are correctly diagnosed as not depressed) [63]. Yet, self-reports are regularly adopted to assess the presence and severity of postnatal depression, and EPDS is among the most sensitive tools [63,64,65].

Finally, the current study focused on the relation of mother and child attachment with self-reported depressive symptoms and parenting stress, excluding the relevance of many other bio-psycho-social factors that inevitably contribute to qualify maternal mental health and the quality of the mother’s attachment relationship to the child [66].

## 5. Conclusions

Apart the above limitations, our study validates the importance of considering maternal representations of attachment crucial in determining the quality of the caregiving environment; thereby the healthy development of children, despite the presence of other contextual risk in particular, future studies should include the paternal role.

Therefore, early intervention should be attachment-based with the aim of supporting mothers, and improving their caregiving behaviors, thus favoring the child’s security of attachment.

## Figures and Tables

**Table 1:** Associations between 71 mothers’ prenatal AAI classifications and the SSP classifications of their children at 15 months.

Mothers’ AAI Classifications			
Children’s SSP Classifications	Autonomous (F) (N = 49)	Dismissing (Ds) (N = 13)	Preoccupied (E) (N = 9)
Secure (B) (N = 52)	41 (57.7%)	4 (5.6%)	7 (9.9%)
Avoidant (A) (N = 7)	3 (4.2%)	3 (4.2%)	1 (1.4%)
Ambivalent (C) (N = 12)	5 (7%)	5 (7%)	2 (2.8%)

**Table 2:** Bivariate intercorrelations among study variables.

	1	2	3	4	5
1. Maternal attachment (1 = secure; 2 = insecure)	_				
2. Child attachment (1 = secure; 2 = insecure)	0.352 **	_			
3. Prenatal depressive symptoms	0.330 **	0.178	_		
4. Postnatal depressive symptoms	0.438 ***	0.295 *	0.600 ***	_	
5. Postnatal parenting stress	0.317 **	0.318 **	0.398 **	0.710 ***	_

Note: * *p* < 0.05; ** *p* < 0.01. *** *p* < 0.001.

**Table 3:** Means and standard deviations for EPDS and PSI-SF scores.

	Mothers’ Attachment Classification		Children’s Attachment Classification	
	Secure (N = 49)	Insecure (N = 22)	Secure (N = 52)	Insecure (N = 19)
	Mean score (SD)	Mean score (SD)	Mean score (SD)	Mean score (SD)
Prenatal depressive symptoms	4.57 (3.34)	7.05 (3.98)	4.83 (3.32)	6.74 (4.45)
Postnatal depressive symptoms	3.55 (3.07)	6.36 (3.08)	3.88 (3.20)	5.89 (3.26)
Postnatal parenting stress	52.78 (9.76)	59.50 (13.17)	52.38 (8.84)	61.63 (14.37)

**Table 4:** Summary of logistic regression analysis predicting child’s attachment at Time 3 (N = 71).

	B	SE	Wald	_*e*B_
Maternal attachment (1 = secure; 2 = insecure)	−1.327	0.651	4.149	0.265 *
Prenatal depressive symptoms	0.019	0.094	0.041	1.019
Postnatal depressive symptoms	−0.028	0.128	0.047	0.973
Postnatal parenting stress	0.072	0.035	4.147	1.074 *
Constant	−4.207	1.853	5.158	0.015 *

Note: * *p* < 0.05.

## Data Availability

The data that support the findings of this study are available on request from the corresponding author.

## References

[1] Ko J.Y., Rockhill K.M., Tong V.T., Morrow B., Farr S.L. (2017). Trends in Postpartum Depressive Symptoms—27 States, 2004, 2008, and 2012. MMWR. Morb. Mortal. Wkly. Rep..

[2] O’Hara M.W., Wisner K. (2013). Perinatal mental illness: Definition, description and aetiology. Best Pract. Res. Clin. Obstet. Gynaecol..

[3] Bernard K., Nissim G., Vaccaro S., Harris J.L., Lindhiem O. (2018). Association between maternal depression and maternal sensitivity from birth to 12 months: A meta-analysis. Attach. Hum. Dev..

[4] Beebe B., Jaffe J., Buck K., Chen H., Cohen P., Feldstein S., Andrews H. (2008). Six-week postpartum maternal depressive symptoms and 4-month mother-infant self- and interactive contingency. Infant Ment. Health J..

[5] Field T. (2010). Postpartum depression effects on early interactions, parenting, and safety practices: A review. Infant Behav. Dev..

[6] Goodman S.H., Brand S.R. (2009). Infants of depressed mothers. Handbook of Infant Mental Health.

[7] Lovejoy M., Graczyk P.A., O’Hare E., Neuman G. (2000). Maternal depression and parenting behavior: A meta-analytic review. Clin. Psychol. Rev..

[8] Cicchetti D., Toth S., Rogosch F.A. (1999). The efficacy of toddler-parent psychotherapy to increase attachment security in offspring of depressed mothers. Attach. Hum. Dev..

[9] Crockenberg S.C., Leerkes E.M. (2003). Parental acceptance, postpartum depression, and maternal sensitivity: Mediating and moderating processes. J. Fam. Psychol..

[10] Martins C., Gaffan E.A. (2000). Effects of Early Maternal Depression on Patterns of Infant-Mother Attachment: A Meta-Analytic Investigation. J. Child Psychol. Psychiatry.

[11] McMahon C., Barnett B., Kowalenko N., Tennant C. (2005). Psychological factors associated with persistent postnatal depression: Past and current relationships, defence styles and the mediating role of insecure attachment style. J. Affect. Disord..

[12] Pallini S., Chirumbolo A., Morelli M., Baiocco R., Laghi F., Eisenberg N. (2018). The relation of attachment security status to effortful self-regulation: A meta-analysis. Psychol. Bull..

[13] Sirois M.-S., Bernier A. (2018). Mother–Child Relationships and Children’s Psychosocial Functioning: The Specific Roles of Attachment Security and Maternal Behavior. Parenting.

[14] Susan Curry J., Krist A.H., Owens D.K., Barry M.J., Caughey A.B., Davidson K.W., Doubeni C.A., Epling J.W., Grossman D.C., US Preventive Services Task Force (2019). Interventions to prevent perinatal depression: US Preventive Services Task Force recommendation statement. JAMA.

[15] Dau A.L.B.B., Callinan L.S., Smith M.V., Dau A.L.B.B., Callinan L.S., Smith M.V. (2019). An examination of the impact of maternal fetal attachment, postpartum depressive symptoms and parenting stress on maternal sensitivity. Infant Behav. Dev..

[16] Ulmer-Yaniv A., Djalovski A., Priel A., Zagoory-Sharon O., Feldman R. (2018). Maternal depression alters stress and immune biomarkers in mother and child. Depress. Anxiety.

[17] Leigh B., Milgrom J. (2008). Risk factors for antenatal depression, postnatal depression and parenting stress. BMC Psychiatry.

[18] Abidin R.R. (1992). The Determinants of Parenting Behavior. J. Clin. Child Psychol..

[19] Deater-Deckard K. (2004). Parenting Stress.

[20] Chang Y., Fine M.A., Ispa J., Thornburg K.R., Sharp E., Wolfenstein M. (2004). Understanding Parenting Stress Among Young, Low-income, African-American, First-Time Mothers. Early Educ. Dev..

[21] Diener M.L., Casady M.A., Wright C. (2003). Attachment Security Among Mothers and Their Young Children Living in Poverty: Associations with Maternal, Child, and Contextual Characteristics. Merrill-Palmer Q..

[22] Ispa J.M., Su-Russell C., Palermo F., Carlo G. (2017). The interplay of maternal sensitivity and toddler engagement of mother in predicting self-regulation. Dev. Psychol..

[23] Shaver P.R., Mikulincer M. (2007). Adult Attachment Strategies and the Regulation of Emotion. Handbook of Emotion Regulation.

[24] Moreira H., Gouveia M.J., Carona C., Silva N., Canavarro M.C. (2015). Maternal Attachment and Children’s Quality of Life: The Mediating Role of Self-compassion and Parenting Stress. J. Child Fam. Stud..

[25] Nygren M., Carstensen J., Ludvigsson J., Frostell A.S. (2012). Adult attachment and parenting stress among parents of toddlers. J. Reprod. Infant Psychol..

[26] Goodman S.H., Rouse M.H., Connell A.M., Broth M.R., Hall C.M., Heyward D. (2011). Maternal Depression and Child Psychopathology: A Meta-Analytic Review. Clin. Child Fam. Psychol. Rev..

[27] Bifulco A., Moran P.M., Ball C., Lillie A. (2002). Adult attachment style. II: Its relationship to psychosocial depressive-vulnerability. Soc. Psychiatry Psychiatr. Epidemiol..

[28] Huth-Bocks A.C., Hughes H.M. (2008). Parenting Stress, Parenting Behavior, and Children’s Adjustment in Families Experiencing Intimate Partner Violence. J. Fam. Violence.

[29] Johansson M., Nordström T., Svensson I. (2021). Depressive symptoms, parental stress, and attachment style in mothers and fathers two and a half years after childbirth: Are fathers as affected as mothers?. J. Child Health Care.

[30] Mason Z., Briggs R., Silver E. (2011). Maternal attachment feelings mediate between maternal reports of depression, infant social–emotional development, and parenting stress. J. Reprod. Infant Psychol..

[31] Nonnenmacher N., Noe D., Ehrenthal J.C., Reck C. (2016). Postpartum bonding: The impact of maternal depression and adult attachment style. Arch. Women’s Ment. Health.

[32] Barnes J., Theule J. (2019). Maternal depression and infant attachment security: A meta-analysis. Infant Ment. Health J..

[33] George C., Kaplan N., Main M. (1996). Adult Attachment Interview.

[34] Main M., Goldwyn R. (1998). Adult Attachment Scoring and Classification System.

[35] Hesse E. (1999). The adult attachment interview: Historical and current perspectives. Handbook of Attachment: Theory, Research, and Clinical Applications.

[36] Cox J.L., Holden J.M., Sagovsky R. (1987). Detection of Postnatal Depression. Development of the 10-Item Edinburgh Postnatal Depression Scale. Br. J. Psychiatry.

[37] Abidin R.R. (1995). Parenting Stress Index: Manual.

[38] Ainsworth M.D.S. (2015). Patterns of Attachment: A Psychological Study of the Strange Situation.

[39] Cassibba R., Coppola G., Sette G., Curci A., Costantini A. (2017). The transmission of attachment across three generations: A study in adulthood. Dev. Psychol..

[40] Verhage M.L., Schuengel C., Madigan S., Fearon R.M.P., Oosterman M., Cassibba R., Bakermans-Kranenburg M.J., van Ijzendoorn M.H. (2016). Narrowing the transmission gap: A synthesis of three decades of research on intergenerational transmission of attachment. Psychol. Bull..

[41] Belsky J. (2005). Attachment Theory and Research in Ecological Perspective: Insights from the Pennsylvania Infant and Family Development Project and the NICHD Study of Early Child Care. Attachment from Infancy to Adulthood: The Major Longitudinal Studies.

[42] Fearon R.P., Belsky J., Cassidy J., Shaver P. (2018). Precursors of attachment security. Handbook of Attachment Theory, Research, and Clinical Applications.

[43] Flykt M., Kanninen K., Sinkkonen J., Punamäki R.-L. (2010). Maternal depression and dyadic interaction: The role of maternal attachment style. Infant Child Dev..

[44] McMahon C.A., Barnett B., Kowalenko N.M., Tennant C.C. (2006). Maternal attachment state of mind moderates the impact of postnatal depression on infant attachment. J. Child Psychol. Psychiatry.

[45] Herrera E., Reissland N., Shepherd J. (2004). Maternal touch and maternal child-directed speech: Effects of depressed mood in the postnatal period. J. Affect. Disord..

[46] Rholes W.S., Simpson J., Friedman M. (2006). Avoidant Attachment and the Experience of Parenting. Pers. Soc. Psychol. Bull..

[47] Lionetti F., Pastore M., Barone L. (2015). Parenting Stress: The Roles of Attachment States of Mind and Parenting Alliance in the Context of Adoption. Parent. Sci. Pract..

[48] George C., Solomon J. (1999). Attachment and caregiving: The caregiving behavioral system. Handbook of Attachment: Theory, Research, and Clinical Applications.

[49] Vismara L., Sechi C., Lucarelli L. (2020). Reflective parenting home visiting program: A longitudinal study on the effects upon depression, anxiety and parenting stress in first-time mothers. Heliyon.

[50] Carlson E.A., Sampson M.C., Sroufe L.A. (2003). Implications of Attachment Theory and Research for Developmental-Behavioral Pediatrics. J. Dev. Behav. Pediatr..

[51] Coyl D.D., Roggman L.A., Newland L.A. (2002). Stress, maternal depression, and negative mother-infant interactions in relation to infant attachment. Infant Ment. Health J..

[52] DeMulder E.K., Denham S., Schmidt M., Mitchell J. (2000). Q-sort assessment of attachment security during the preschool years: Links from home to school. Dev. Psychol..

[53] Jarvis P.A., Creasey G.L. (1991). Parental stress, coping, and attachment in families with an 18-month-old infant. Infant Behav. Dev..

[54] Tharner A., Luijk M.P.C.M., Van Ijzendoorn M.H., Bakermans-Kranenburg M.J., Jaddoe V.W.V., Hofman A., Verhulst F.C., Tiemeier H. (2012). Infant Attachment, Parenting Stress, and Child Emotional and Behavioral Problems at Age 3 Years. Parent. Sci. Pract..

[55] Anthony L.G., Anthony B.J., Glanville D.N., Naiman D.Q., Waanders C., Shaffer S. (2005). The relationships between parenting stress, parenting behaviour and preschoolers’ social competence and behaviour problems in the classroom. Infant Child Dev..

[56] Dubber S., Reck C., Müller M., Gawlik S. (2015). Postpartum bonding: The role of perinatal depression, anxiety and maternal–fetal bonding during pregnancy. Arch. Women’s Ment. Health.

[57] Toth S.L., Rogosch F.A., Sturge-Apple M., Cicchetti D. (2009). Maternal Depression, Children’s Attachment Security, and Representational Development: An Organizational Perspective. Child Dev..

[58] Śliwerski A., Kossakowska K., Jarecka K., Świtalska J., Bielawska-Batorowicz E. (2020). The Effect of Maternal Depression on Infant Attachment: A Systematic Review. Int. J. Environ. Res. Public Health.

[59] Monk C., Leight K.L., Fang Y. (2008). The relationship between women’s attachment style and perinatal mood disturbance: Implications for screening and treatment. Arch. Women’s Ment. Health.

[60] Netsi E., Pearson R.M., Murray L., Cooper P., Craske M.G., Stein A. (2018). Association of Persistent and Severe Postnatal Depression with Child Outcomes. JAMA Psychiatry.

[61] Stein A., Pearson R., Goodman S.H., Rapa E., Rahman A., McCallum M., Howard L., Pariante C.M. (2014). Effects of perinatal mental disorders on the fetus and child. Lancet.

[62] Vliegen N., Casalin S., Luyten P. (2013). The course of postpartum depression: A review of longitudinal studies. Harv. Rev. Psychiatry.

[63] Gaynes B.N., Gavin N., Meltzer-Brody S., Lohr K.N., Swinson T., Gartlehner G., Brody S., Miller W.C. (2005). Perinatal Depression: Prevalence, Screening Accuracy, and Screening Outcomes. Evid. Rep. Technol. Assess..

[64] Gollan J.K., Mesches G.A., Gortner I.A., Martin C., Hunter L.A., Patel V., Preedy V., Rajendram R. (2021). Edinburgh postnatal depression scale: Description and applications. The Neuroscience of Depression.

[65] Graffi J., Moss E., Jolicoeur-Martineau A., Moss G., Lecompte V., Pascuzzo K., Babineau V., Gordon-Green C., Mileva-Seitz V.R., Minde K. (2018). The dopamine D4 receptor gene, birth weight, maternal depression, maternal attention, and the prediction of disorganized attachment at 36 months of age: A prospective gene × environment analysis. Infant Behav. Dev..

[66] Measelle J.R., Ablow J. (2018). Contributions of early adversity to pro-inflammatory phenotype in infancy: The buffer provided by attachment security. Attach. Hum. Dev..

